# Congenital muscular ventricular septal aneurysm coexisting with a pericardial cyst, a case report

**DOI:** 10.1186/s43044-026-00786-9

**Published:** 2026-09-28

**Authors:** Qian Mou, Jin-jing Li, Xue Li, Qiang Chen

**Affiliations:** Shenzhen Hospital of Southern Medical University, Shenzhen, China

**Keywords:** Muscular ventricular septal aneurysm, Pericardial cyst, Heart failure, Case report

## Abstract

**Background:**

Muscular ventricular septal aneurysm (VSA) is commonly caused by myocardial infarction, myocarditis or trauma, but it may occasionally be congenital. For congenital VSA, the diagnosis is challenging, and the therapeutic strategy and prognosis remain uncertain.

**Case presentation:**

A 32-year-old man was admitted following a detection of left ventricular aneurysm on echocardiography during a routine health checkup. The patient was asymptomatic, the echocardiography showed an aneurysm in the midventricular septum associated with regional hypokinesis, accompanied by left ventricular dilation and reduced left ventricular ejection fraction (LVEF) of 46%. Coronary computed tomography angiography and cardiac magnetic resonance localized the muscular VSA and revealed a cyst at the anteroinferior margin of the pericardium, further coronary angiography was unremarkable. Laboratory findings such as cardiac markers, rheumatologic, immunologic and inflammatory markers were within normal limits. The diagnosis of congenital muscular VSA coexisting with a pericardial cyst was considered. Pharmacological treatment for heart failure was initiated to prevent further reduction in LVEF. At the 3-month and 1-year follow-ups the patient remained asymptomatic, and echocardiography demonstrated the LVEF increased from 50% to 57%.

**Conclusion:**

Congenital muscular VSA is an uncommon cardiac abnormality that may be accompanied by impaired cardiac function. Treatment for heart failure could improve the cardiac function.

## Background

Ventricular septal aneurysm (VSA) is commonly caused by myocardial infarction, myocarditis, or trauma [1, 2], which lead to regional myocardial necrosis and remodeling. However, this structural abnormality may also be congenital according to limited literature reports [3, 4], presenting with a variety of clinical manifestations [5].

## Case presentation

**Fig. 1:**
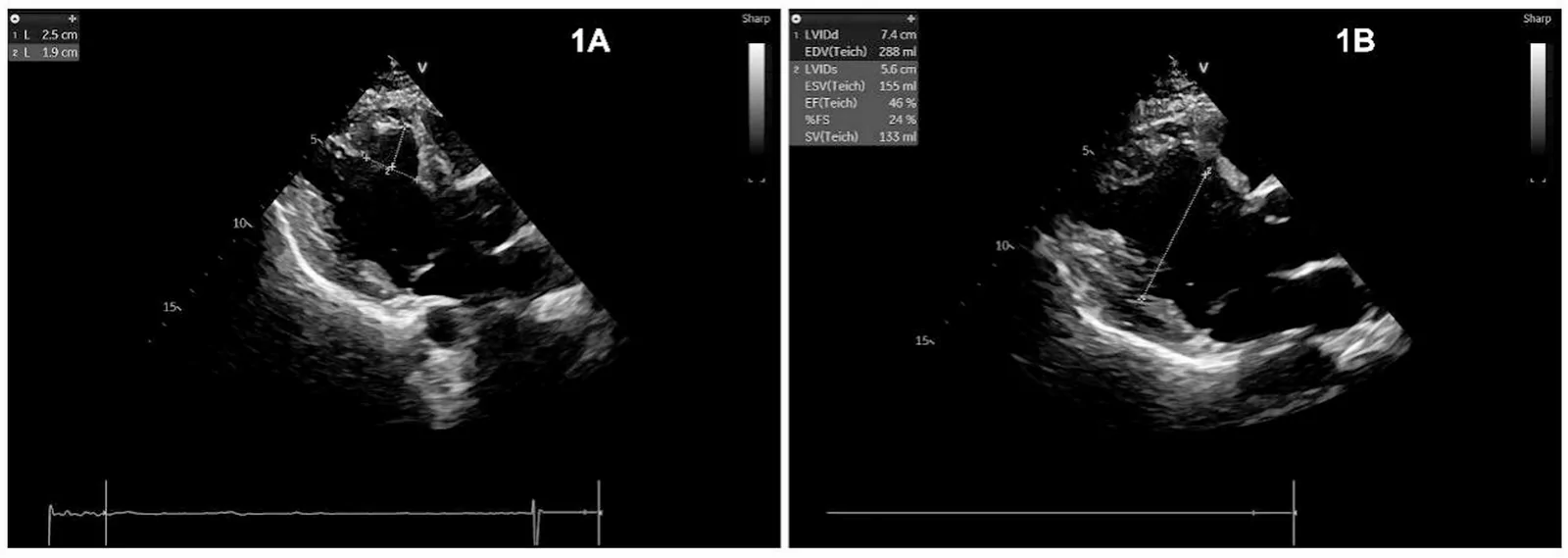
Echocardiography demonstrated a muscular ventricular septal aneurysm. 1 **A**. Location and size of the aneurysm. 1 **B**. The calculated left ventricular function

A 32-year-old man was admitted following a detection of “a 25 mm* 19 mm aneurysm in the midventricular septum” on echocardiography during a routine health check-up (Fig. 1).

**Fig. 2:**
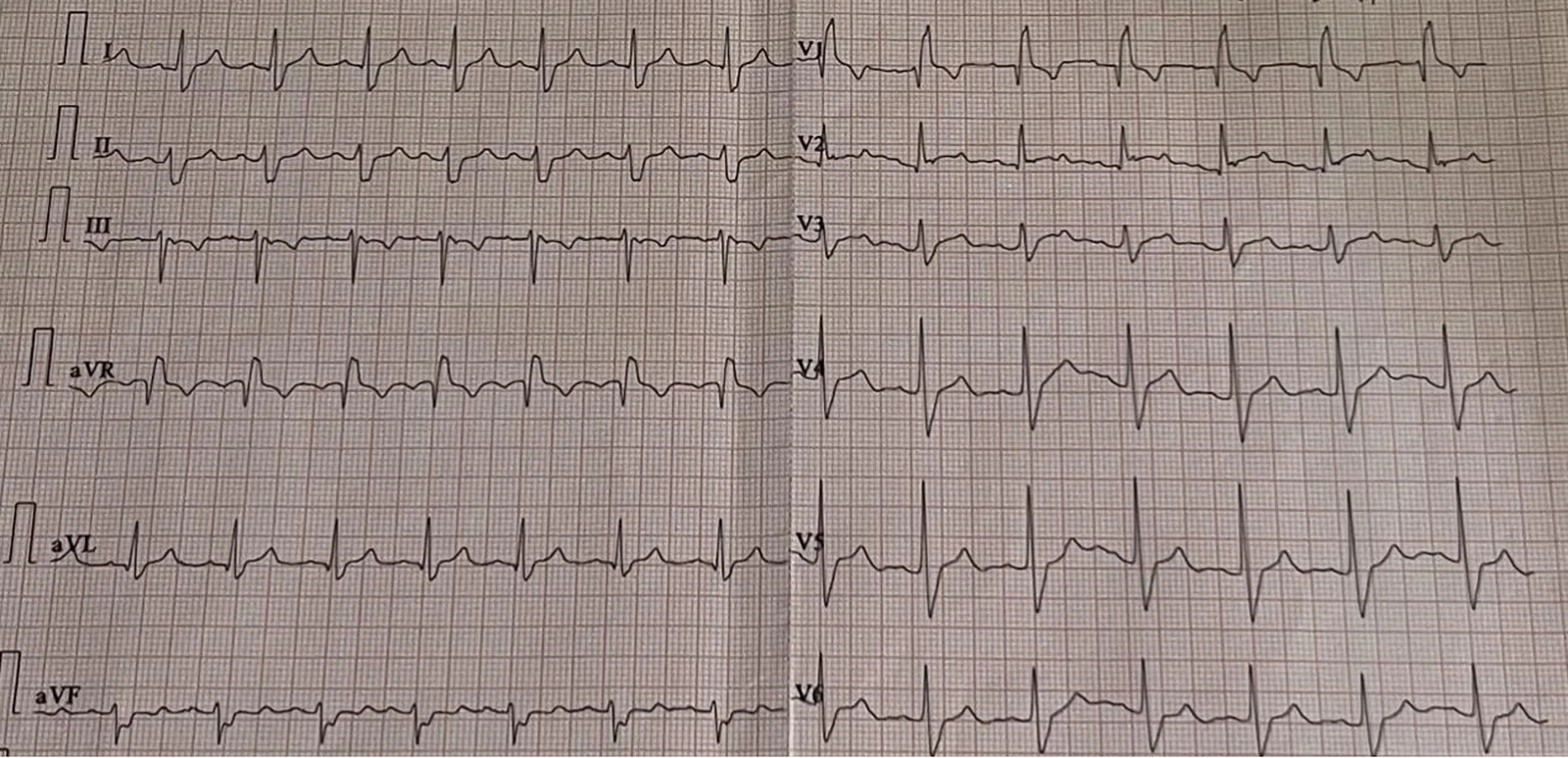
Electrocardiogram showed sinus rhythm with complete right bundle branch block

Additional echocardiographic findings included regional hypokinesis of anterior wall and left ventricular enlargement, end diastolic volume (EDV) and end systolic volume (ESV) were 175 mL and 94 mL, respectively. The calculated left ventricular ejection fraction (LVEF) reduced to 46% by Simpson’s method. On admission, the patient was asymptomatic and reported no significant medical history, auscultation of heart and lungs was unremarkable. Cardiac markers (NT-proBNP, high-sensitivity troponin I), rheumatologic, immunologic and inflammatory markers were within normal limits. Electrocardiogram (ECG) showed sinus rhythm with complete right bundle branch block (RBBB) (Fig. 2).

**Fig. 3:**
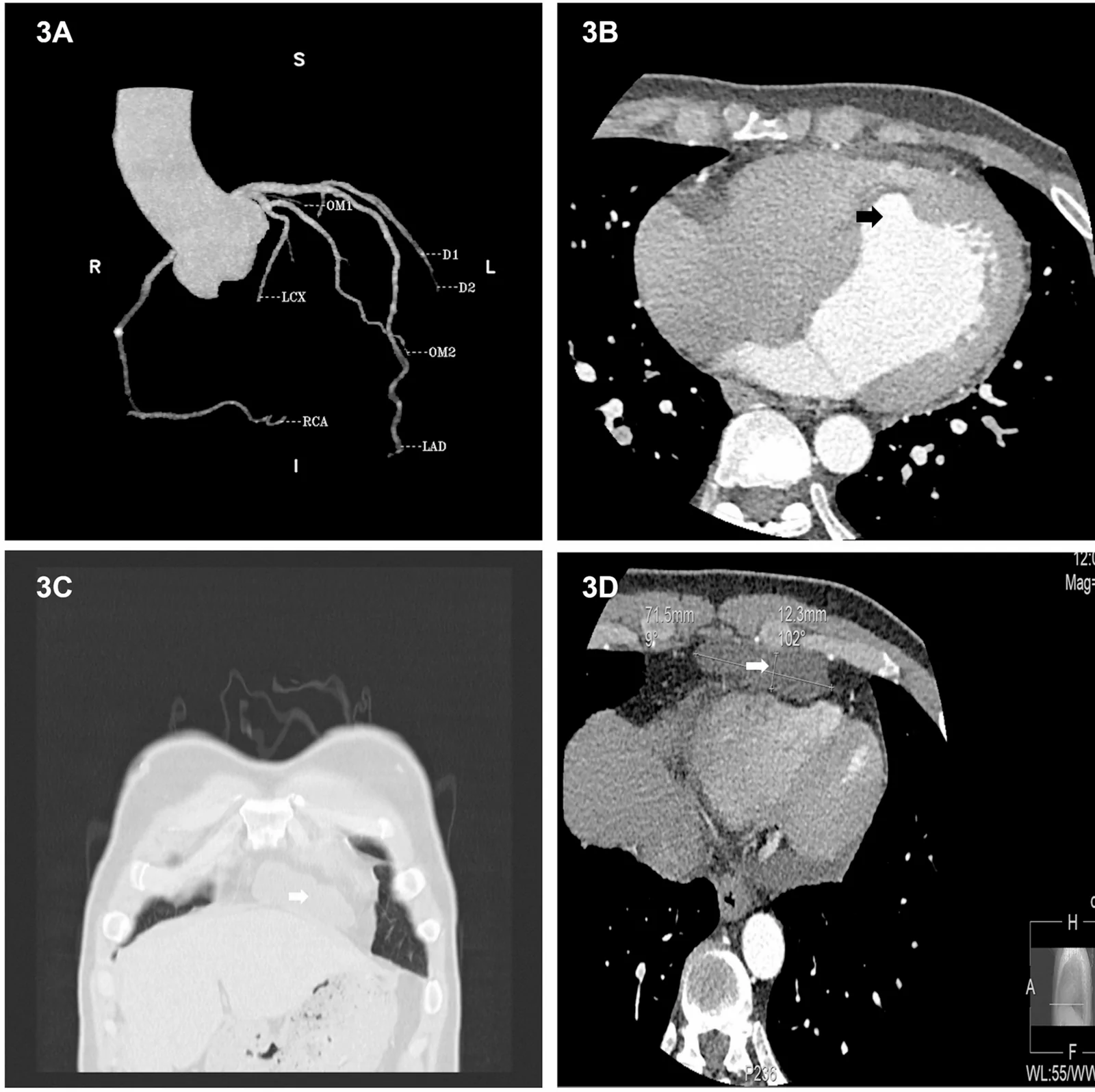
The presentations of coronary computed tomography angiography. 3 **A**. Normal coronary presentation. 3 **B**. A muscular ventricular septal aneurysm with focal fatty replacement (black arrow). 3 **C** &3 **D**. A cyst located in the anteroinferior margin of the pericardium (white arrow)

The coronary computed tomography angiography demonstrated a normal coronary presentation, as well as a muscular aneurysm with focal fatty replacement in the midventricular septum. Besides, a 72 mm*12 mm well-defined, thin-walled cystic lesion was identified at the anteroinferior margin of the pericardium. (Fig. 3).

**Fig. 4:**
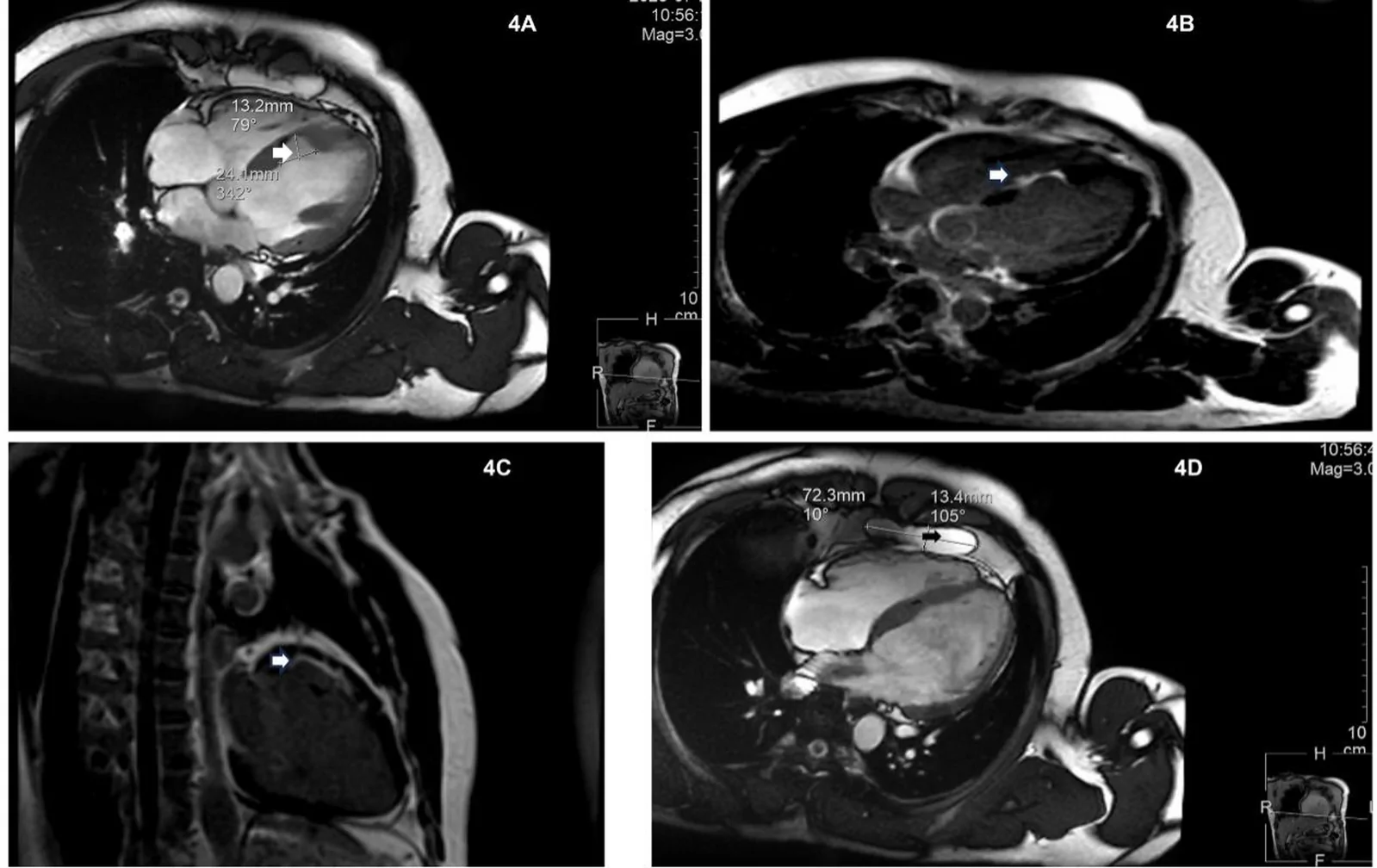
The presentations of cardiac magnetic resonance. 4 **A**. An aneurysm in muscular ventricular septum (white arrow). 4 **B** & 4 **C**. Delayed gadolinium enhancement established the extent of fibrosis within the aneurysm (white arrow). 4 **D**. A thin-walled cyst was adjacent to the pericardium (black arrow)

The cardiac magnetic resonance (CMR) provided a superior imaging evaluation. The muscular VSA measured about 24 mm * 13 mm with an intact septal structure, delayed gadolinium enhancement (LGE) demonstrated the extent of fibrosis within the aneurysm (Fig. 4). Hypokinesis of the ventricular septum and basal anterior wall was documented, the EDV and ESV were 249mL and 154mL, respectively, the LVEF was 38% calculated by Simpson’s method. A 72 mm *13 mm thin-walled cyst was adjacent to the pericardium but did not communicate directly with it, suggesting the diagnosis of pericardial cyst.

**Fig. 5:**
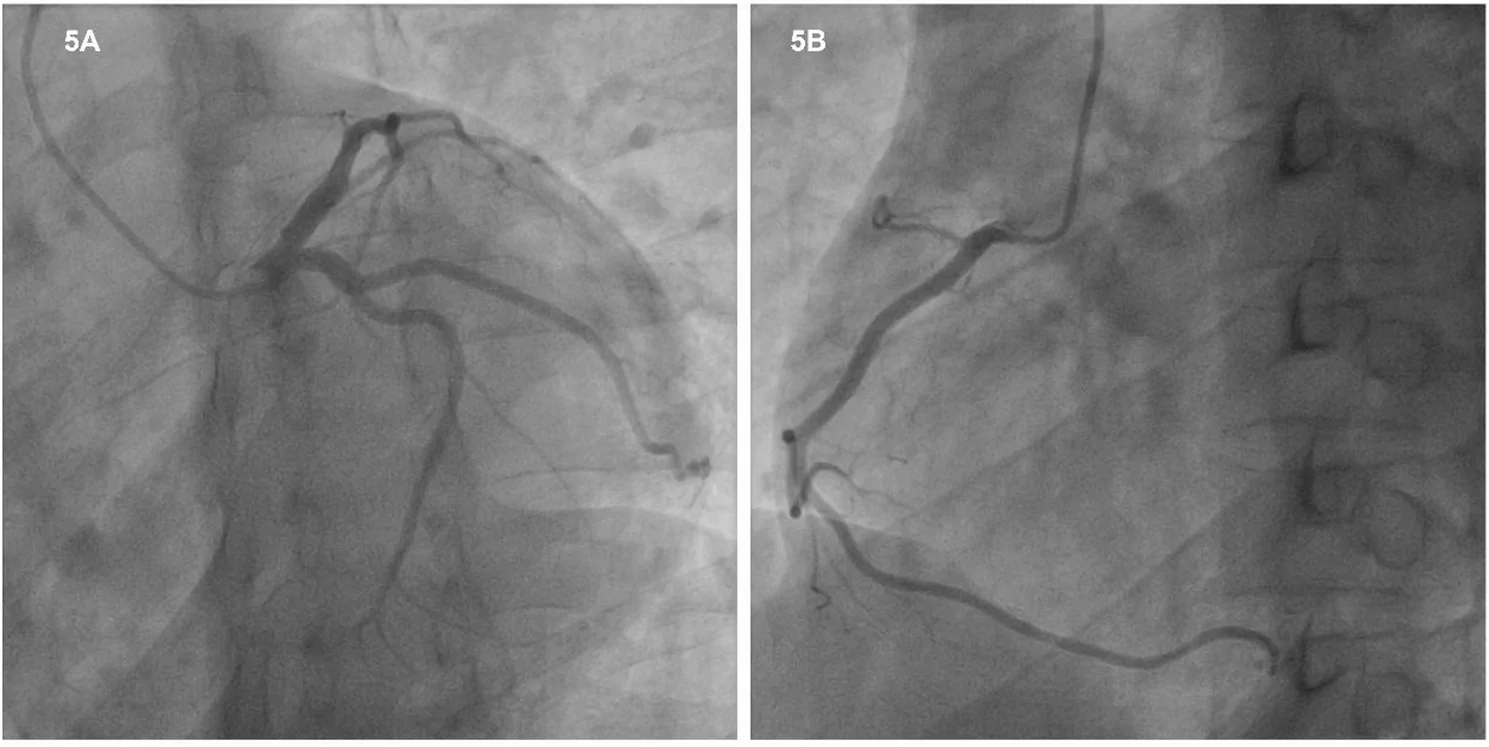
Coronary angiography demonstrated normal coronary presentation. 5 **A**. Normal left coronary artery. 5 **B**. Normal right coronary artery

Although coronary CT showed no coronary anomalies, microvascular dysfunction such as slow flow or coronary vasospasm could not be excluded. Subsequent coronary angiography demonstrated smooth coronaries with normal TIMI grade 3 flow, without evidence of vasospasm (Fig. 5). To date, the available clinical findings did not support the diagnosis of ischemic heart disease, myocarditis, inflammatory or immunologic-related disease, we therefore concluded that the muscular VSA coexisting with a pericardial cyst were of congenital origin.

A surgical consultation was obtained, conservative treatment and long-term follow-up were recommended. Base on reduction of LVEF, the patient was prescribed medications for heart failure, including angiotensin-converting enzyme inhibitor, beta-blocker and sodium-glucose cotransporter 2 inhibitor. So far, the 3-month and 1-year follow-ups were arranged, the patient remained asymptomatic, with no change on ECG. The 3-month echocardiography showed the aneurysm measured 27*16 mm, with EDV and ESV of 184 mL and 92 mL, respectively. The LVEF increased to 50%. The 1-year echocardiography revealed the aneurysm measured 25*14 mm, with improved anterior wall motion, with EDV and ESV of 155 mL and 66 mL, respectively. The LVEF showed further improvement to 57%. The patient continued previous medications and was scheduled for further follow-up.

## Discussion

Muscular VSA is very uncommon, the main diagnostic challenge is to distinguish between congenital abnormality [3, 4] and acquired heart diseases, such as myocardial infarction, cardiomyopathies, myocarditis and trauma [1, 2]. In this case, young age without ischemic risk factors, normal coronary presentation, normal laboratory results of rheumatologic, immunologic and inflammatory markers supported the diagnosis of congenital muscular VSA.

Congenital muscular VSA has been reported in both children and adults [5, 6], it may occur as an isolated lesion or in association with atrial septal defect, patent ductus arteriosus or other congenital defects [5]. Conduction abnormality, Wolf-Parkinson-White syndrome [6, 7] and other cardiac arrhythmias have been reported previously. RBBB was observed in this case and remained unchanged during follow-up, however, the clinical significance remains unclear, and routine ECG should be performed in the subsequent follow-ups.

Therapeutic strategy for congenital VSA remains uncertain, as clinical manifestations vary from asymptomatic presentation to heart failure [4, 5], suggesting that the prognosis may be unfavorable [3]. In this case, reduced left ventricular function was observed by echocardiography and CMR. As a result, we initiated pharmacological treatment for heart failure. Encouragingly, echocardiography showed gradual improvement in cardiac function at the 3-month and 1-year follow-ups.

As far as the pericardial cyst is concerned, a congenital anomaly is more common than an acquired lesion [8]. The clinical presentation depends on the morphology and location of the cyst and varies from asymptomatic status to compressive symptoms, including dyspnea, chest tightness, and epigastric pain [9]. For symptomatic patients, percutaneous aspiration or surgical removal of the cyst are recommended. For patients without symptoms, conservative management with routine follow-up appears to be optimal and easily accepted [10]. In this case, the patient was asymptomatic, his previous medical history and diagnostic workup were unremarkable, we therefore hypothesized that the pericardial cyst was also congenital. However, the conclusion was drawn based on CT and MRI findings, since cyst specimen was not obtained, the histopathological diagnosis could not be confirmed.

Several limitations should be noticed in this case. First, during coronary angiography, provocative testing, the gold standard for diagnosing coronary vasospasm was not performed. Left ventricular angiogram was also not performed because CMR had been obtained previously. Second, follow-up cardiac functional assessment was carried out by echocardiography rather than by CMR, which could provide more detailed functional information. Third, other imaging evaluation such as cardiac PET-CT was not arranged, it should be considered if cardiac function deteriorated during further follow-ups.

In summary, congenital muscular VSA is a rare cardiac anomaly that might coexist with another congenital lesion such as a pericardial cyst. The treatment strategy and prognosis for congenital muscular VSA remain uncertain, Pharmacological therapy for heart failure should be initiated once evidence for cardiac dysfunction is identified.

## Data Availability

All data generated or analyzed during this study are included in this published article [and its supplementary information files].

## Abbreviations

VSA: Ventricular septal aneurysm
LVEF: Left ventricular ejection fraction
EDV: End diastolic volume
ESV: End systolic volume
RBBB: Right bundle branch block
CMR: Cardiac magnetic resonance
LGE: Delayed gadolinium enhancement
